# Clathrin Heavy Chain Is Important for Viability, Oviposition, Embryogenesis and, Possibly, Systemic RNAi Response in the Predatory Mite *Metaseiulus occidentalis*

**DOI:** 10.1371/journal.pone.0110874

**Published:** 2014-10-20

**Authors:** Ke Wu, Marjorie A. Hoy

**Affiliations:** Department of Entomology and Nematology, University of Florida, Gainesville, Florida, United States of America; Ghent University, Belgium

## Abstract

Clathrin heavy chain has been shown to be important for viability, embryogenesis, and RNA interference (RNAi) in arthropods such as *Drosophila melanogaster*. However, the functional roles of clathrin heavy chain in chelicerate arthropods, such as the predatory mite *Metaseiulus occidentalis,* remain unknown. We previously showed that dsRNA ingestion, followed by feeding on spider mites, induced systemic and robust RNAi in *M. occidentalis* females. In the current study, we performed a loss-of-function analysis of the *clathrin heavy chain* gene in *M. occidentalis* using RNAi. We showed that ingestion of *clathrin heavy chain* dsRNA by *M. occidentalis* females resulted in gene knockdown and reduced longevity. In addition, *clathrin heavy chain* dsRNA treatment almost completely abolished oviposition by *M. occidentalis* females and the few eggs produced did not hatch. Finally, we demonstrated that *clathrin heavy chain* gene knockdown in *M. occidentalis* females significantly reduced a subsequent RNAi response induced by ingestion of *cathepsin L* dsRNA. The last finding suggests that clathrin heavy chain may be involved in systemic RNAi responses mediated by orally delivered dsRNAs in *M. occidentalis*.

## Introduction

Endocytosis, a crucial cellular process in all eukaryotes, is involved in the uptake of nutrients and control of the density of receptors on the cell surface [1], [2], [3]. Many endocytotic processes require protein-coated pits or vesicles, in which clathrin is a major component. Clathrin is involved in coating membranes that are endocytosed from plasma membranes and those that move between the *trans*-Golgi network and endosomes [4]. Three clathrin heavy chains (∼190 kDa each), each bound by a clathrin light chain, are joined at their carboxy termini to form a basic clathrin subunit. Multimerization of clathrin subunits form a polyhedral protein lattice that is found on clathrin-coated pits and vesicles.

Genetic disruption of the *clathrin heavy chain* gene, which is conserved across eukaryotic species, has facilitated the study of clathrin function in many organisms. In single-cell organisms such as yeast, inactivation of the *clathrin heavy chain* gene resulted in slowed growth or lethality [5], [6]. The amoeba *Dictyostelium discoideum* exhibited reduced growth rates and reduced endocytosis following *clathrin heavy chain* ablation [7]. Disruption of the *clathrin heavy chain* gene in the protozoan *Trypanosoma brucei* resulted in lethality and essentially undetectable endocytosis [8]. A number of knockout and knockdown studies in mammalian cell cultures showed that repression of the *clathrin heavy chain* gene resulted in significantly reduced clathrin-mediated endocytosis of various cargos including transferrin [9], epidermal growth factor, and a LDL receptor chimera [10]. *In vivo*, Bazinet et al. [11] showed that several *clathrin heavy chain* mutants induced by ethyl methanesulfonate were lethal in *Drosophila melanogaste*r. These mutants also displayed embryonic lethality, suggesting that the *clathrin heavy chain* gene was indispensable to embryonic development in this insect. Moreover, ablation of the *clathrin heavy chain* gene in the oocytes of *Caenorhabditis elegans* using RNA interference (RNAi) resulted in dead embryos and reduced yolk uptake [12]. These studies suggest that the highly conserved clathrin heavy chain protein plays an important role in the endocytotic process in many eukaryotes and is likely to be essential for viability and embryogenesis in multicellular organisms.

Clathrin heavy chain has been implicated in the RNAi pathway in several species based on the results of several *in vitro* and *in vivo* studies. *Clathrin heavy chain* gene knockdown by RNAi reduced subsequent RNAi responses in *Drosophila* cell cultures, possibly by blocking the uptake of dsRNA through clathrin-mediated endocytosis (or receptor-mediated endocytosis) [13], [14]. A study in the desert locust *Schistocerca gregaria* showed that injections of dsRNA for the *clathrin heavy chain* gene attenuated a subsequent RNAi response in the midgut, suggesting that the clathrin-mediated endocytosis pathway may be involved in the RNAi response in the midgut of this insect [15]. A study in the tick *Haemaphysalis longicornis* showed that injections of dsRNA for a scavenger receptor reduced subsequent RNAi responses [16]. Because clathrin heavy chain is thought to be involved in all receptor-mediated endocytosis, this result suggests that clathrin heavy chain may play a role in the RNAi responses in ticks. Many arthropods initiate systemic RNAi responses following ingestion of dsRNAs (for a review, see [17]). However, it is unclear what role clathrin heavy chain plays in these RNAi responses.

*Metaseiulus* ( = *Typhlodromus* or *Galendromus) occidentalis* (Nesbitt) (Arthropoda: Chelicerata: Arachnida: Acari: Phytoseiidae) is an agriculturally important biological control agent of plant-feeding pest mites such as *Tetranychus urticae*. Previously, pesticide-resistant strains of *M. occidentalis* were developed through laboratory selection and these genetically improved mites were applied in biological control programs [18], [19], [20]. Further genetic improvement can benefit from studies on the molecular components that are important for the viability and embryonic development in this species. These studies may be performed using reverse genetic tools such as RNAi. We recently showed that orally delivered dsRNA induced robust and systemic RNAi responses in *M. occidentalis* females, but only after spider mite prey was provided [21]. It is unknown whether clathrin heavy chain plays any role in the RNAi response in *M. occidentalis*.

Due to the high degree of sequence homology of clathrin heavy chains across different species, we hypothesized that this protein is important for the viability and embryogenesis of *M. occidentalis*. We also hypothesized that clathrin heavy chain is involved in the RNAi responses in *M. occidentalis* following the ingestion of dsRNA. To test our hypotheses, we first identified a putative *clathrin heavy chain* gene in the *M. occidentalis* genome that was recently sequenced and annotated [Hoy, et al., unpublished]. We then conducted a loss-of-function analysis of the *clathrin heavy chain* gene in *M. occidentalis* females using RNAi. After *clathrin heavy chain* dsRNA was introduced into *M. occidentalis* females through ingestion, we evaluated possible gene knockdown and measured the effects of *clathrin heavy chain* dsRNA ingestion on the longevity and egg production of these females. The hatchability of the eggs produced by these females was evaluated as well. Finally, we evaluated the effects of *clathrin heavy chain* dsRNA delivery on a subsequent RNAi response mediated by ingestion of *M. occidentalis cathepsin L* dsRNA.

## Materials and Methods

### Colony sources and maintenance

The F10A inbred line was derived from the COS (Carbaryl-OP-Sulfur-resistant) colony [22], [23] by sibmating single pairs for 10 generations, as described previously [24]. The F10A colony was maintained and all experiments were performed at 22–23°C and a relative humidity (RH) of 45–55%, under a 16L:8D photoperiod. All stages of *T. urticae* were brushed on to paraffin-coated construction paper (75 mm×75 mm) resting on water-soaked cotton to serve as prey.

Age-matched, mated females for loss-of-function and quantitative reverse transcription-PCR (qRT-PCR) analyses were produced as described previously [21]. Briefly, 20 females of unknown age were collected from the *M. occidentalis* F10A colony and placed on pinto bean (*Phaseolus vulgaris*) leaf discs (40 mm×60 mm) resting on water-soaked cotton that were infested with approximately 50 *T. urticae* females. These females were allowed to lay eggs for one day and were then removed. The eggs produced were allowed to hatch and develop. Seven days later, adult females and males emerged and were allowed to mate. Two days later, gravid (mated) females were collected individually and placed on pinto bean leaf discs (15 mm in diameter) that were infested with 4–5 *T. urticae* females. One day later, these females produced 1–2 eggs/female, indicating that they had mated.

### Identification of putative clathrin heavy chain and cathepsin L genes in the *Metaseiulus occidentalis* genome

The amino-acid sequence of the *D. melanogaster clathrin heavy chain* (accession number: NP_477042.1) gene was used to search for putative homologs in the *M. occidentalis* genome database in GenBank using BLASTP. One hit (accession number XP_003740941.1) was annotated as *clathrin heavy chain* by the NCBI. Similarly, the amino-acid sequence of *D. melanogaster* cysteine proteinase-1 (accession number NP_725347.1) was used to search for putative homologs of cysteine proteinase-1 in the *M. occidentalis* genome. One of the top hits (accession number: XP_003738729.1; BLASTP E value: 3e-126) was identified as a putative homolog of *Drosophila* cysteine proteinase-1 and was annotated as cathepsin L-like protein by the NCBI. BLASTP searches were conducted using the cutoff E value of 1e-5. All searches were performed with the BLAST default settings.

### Annotation of *clathrin heavy chain* domains and phylogenetic analysis

The conserved domains of *clathrin heavy chain* genes were identified by searching the conserved domain database of the NCBI (http://www.ncbi.nlm.nih.gov/Structure/cdd/wrpsb.cgi) using default settings [25] with the amino-acid sequences of *clathrin heavy chain* homologs. An alignment of the deduced amino-acid sequences of *clathrin heavy chain* genes was conducted using MAFFT 7.147 [26] with the E-INS-i alignment algorithm and the BLUSUM 62 matrix. Model selection was done with ProtTest 3.2 [27] and according to the Akaike information criterion, the LG+I+G model was optimum for phylogenetic analysis. Finally, a maximum likelihood analysis was performed using RAxML 7.3.2 [28], bootstrapping with 1,000 replicates.

### RNA extraction and cDNA synthesis

RNA from *M. occidental* females was extracted using RNAqueous-Micro kit (Part Number Am1931, Life Technologies, CA, USA) according to the manufacturer’s instructions. cDNA for cloning was made using the cloned AMV first-strand cDNA synthesis kit (cat. no. 12328-032, Invitrogen, CA, USA) according to the manufacturer’s instructions. Nine µl of RNA isolated from a pooled sample of *M. occidentalis* mites (20 females, 20 males, and 20 eggs, 20 nymphs and 20 larvae) were used in a 20-µl reverse transcription reaction containing the manufacturer’s recommended ingredients including Oligo(dT)_20_ primers. The reaction was performed in a thin-walled tube using a thermocycler (GeneAmp PCR system 9700, Applied Biosystems). The reaction was incubated at 50°C for 60 min, followed by incubation at 85°C for 5 min.

cDNA for qRT-PCR was made using high-capacity cDNA reverse transcription kits (part number 4375575, Applied Biosystems, CA, USA) according to the manufacturer’s instructions. Thirteen µl of RNA from individual *M. occidentalis* females were used in a 20-µl reaction containing all ingredients. The reaction was incubated at 25°C for 10 min, then 37°C for 120 min, and 85°C for 5 min. Sixty µl of TE (10 mM Tris pH 7.5, 1 mM EDTA) was then added to the cDNA reaction (1:4 dilution). cDNA synthesized by both methods was stored at −20°C until use.

### dsRNA synthesis

Primers with the T7 promoter sequence (TAATACGACTCACTATAGGGAG) added at the 5′ end were used to amplify fragments (∼500 bp) of *clathrin heavy chain* and *cathepsin L* genes that were later used for dsRNA synthesis. The sequences for the forward and reverse primers used to amplify *clathrin heavy chain* gene (accession number for putative mRNA: XM_003740893.1) fragments are “TAATACGACTCACTATAGGGAGCGGCACGATGTCGTCTATTT” and “TAATACGACTCACTATAGGGAGCTCCGAACACTCCAACTTCTC”, respectively. The sequences for the forward and reverse primers used to amplify *cathepsin L* gene (accession number for putative mRNA: XM_003738681.1) fragments are “TAATACGACTCACTATAGGGAGCAAGCTCGTCTCGCTTTCT” and “TAATACGACTCACTATAGGGAG CTGGTTCTCCTTGTTCCTCATC”, respectively. PCR products were amplified from 1 µl of cDNA prepared from a pooled sample of 20 *M. occidentalis* females, males, eggs, nymphs and larvae using procedures described previously [21]. Bands of the expected size (500 bp) were extracted and purified using a Gel Extraction kit (Qiagen, Valencia, California) according to the manufacturer’s protocol. Direct sequencing of the purified PCR products was performed at the Interdisciplinary Center for Biotechnology Research at the University of Florida using the primers used for PCR amplification. dsRNAs were synthesized from 1 µg of purified PCR products or control template provided by the MEGAscript RNAi kit (cat. No. AM1626, Life technologies, CA, USA) according to the manufacturer’s instructions. Sizes of purified dsRNAs were confirmed by gel electrophoresis in 1% agarose gel containing TBE buffer. Concentrations of purified dsRNAs were determined by a spectrophotometer (NanoDrop 1000, Thermo Scientific, USA). Purified dsRNAs were stored in elution buffer at 20°C until further use.

### dsRNA ingestion

Ingestion of dsRNA was performed as described previously [21]. Briefly, 10–20 age-matched F10A mated females were starved for 24 h and then placed on a parafilm disc (22 mm in diameter) resting on water-soaked cotton at 22–23°C, 45–55% RH and 16L:8D photoperiod. Ten µl of solution containing 350 ng dsRNA/µl in 20% sucrose (Sigma) and with 3% blue food dye (McCormick, MD, USA) was applied to the parafilm disc. Both sucrose solution and blue food dye were boiled for 10 min to eliminate potential contamination by nucleases before use. F10A females were allowed to feed on the dsRNA/sucrose solution for 48 h. Ingestion was confirmed by the presence of intense blue color in the gastric caecae. During the current experiment, it took “starved” F10A females between 2–12 hrs to start ingesting the dsRNA/20% sucrose solution by which time the dsRNA/sucrose droplets had lost volume due to evaporation. The dsRNA/sucrose droplets kept losing volume up to 16 hrs, indicating that sucrose concentrations in the droplets reached a saturated level. A lower concentration (i.e. 10%) of sucrose was evaluated for feeding experiments previously and dsRNA/10% sucrose droplets experienced a greater loss of volume than dsRNA/20% sucrose droplets (data not shown). Therefore, to maintain the dsRNA concentrations at a more consistent level during the feeding period, 20% sucrose solutions were used for subsequent feeding experiments. Efforts to reduce the loss of dsRNA/20%sucrose volume by conducting feeding experiments in a high relative humidity chamber (∼90% RH, ∼23°C) only resulted in more variability in the amounts of food dye (a marker for ingested dsRNA/sucrose) present in different females (data not shown). Therefore, feedings of dsRNA were performed using the conditions specified above. Only females with blue dye within the caecae were used for further analyses. Less than 10% of the females were lost due to runoff. Mated females deposited no eggs during the starvation and dsRNA ingestion periods, consistent with the fact that *M. occidentalis* is an obligatory predator that requires feeding on prey to oviposit.

### Loss-of-function analysis

To measure the effects of *clathrin heavy chain* gene knock down on viability and embryogenesis, females of *M. occidentalis* that ingested control dsRNA in sucrose, *clathrin heavy chain* dsRNA in 20% sucrose, and TE in sucrose were collected individually and placed, with a virgin male, on a bean leaf disc (22 mm in diameter) on water-soaked cotton that had been infested for 24 h with 10 *T. urticae* females (in order to produce prey eggs). Ingestion of prey was indicated by an increase in female body size and gradual disappearance of blue color from the gastric caecae [data not shown]. Oviposition started ∼48 h after the spider mite diet was provided. The eggs produced by the *M. occidentalis* females were counted and collected every day with a fine sable-hair brush and transferred individually to a new bean leaf disc (22 mm in diameter) containing 4 *T. urticae* females per *M. occidentalis* egg. The hatch rate of eggs deposited by *M. occidentalis* females, as well as the day of death for each female, was recorded. For the analysis of *clathrin heavy chain* gene knockdown, 2 days after being provided with spider mite prey, 4 *M. occidentalis* females that were fed with control or *clathrin heavy chain* dsRNA were collected and total RNA from individual females was extracted. qRT-PCR was performed subsequently to determine the *clathrin heavy chain* mRNA levels.

To evaluate the effects of *clathrin heavy chain* gene knockdown on subsequent RNAi responses, 40 age-matched F10A females were divided into 2 groups that were fed *clathrin heavy chain* or control dsRNAs, as described above. These females were provided *T. urticae* prey for 2 days and then starved for 1 day. Half of the females from each group were provided with control dsRNA and the other half were provided with *cathepsin L* dsRNA for 2 days. All females were then provided with *T. urticae* prey for 2 days before being collected for RNA extractions and subsequent qRT-PCR. Approximately 50% of females survived the assay period.

### qRT-PCR analysis

qRT-PCR analysis was performed in a similar manner as described previously [21]. The sequences for the forward and reverse primers used for the qRT-PCR of *clathrin heavy chain* gene are “GGAGCACTACACGGATCTTTAC” and “AGTGAGTCTTCAACCGAAAGG”, respectively. The sequences for the forward and reverse primers used for the qRT-PCR of *cathepsin L* gene are “CTTCAGACGTCAGCTGTTCTAC” and “GAACTCCTCGCTGGTCATATC”, respectively. The sequences for the forward and reverse primers used for the qRT-PCR of *actin* gene (internal control; accession number for putative mRNA: XM_003742554.1) are “ACATCAAGGAGAAGCTCTGC” and “CCTCGGGACAACGGAAAC”, respectively. The primers were validated using standard curves based on serial dilutions of cDNA to determine the primer annealing efficiencies. One no-template control was included in each experiment to check for possible contamination. qRT-PCR (in technical triplicates) was performed in a 20-µL reaction volume containing 1 µL of cDNA, 0.5 µL of forward and reverse primers (8 µM each), 10 µl of SYBR select master mix (Applied Biosystems), and 8 µl of nuclease-free H_2_O. To minimize potential variations in qRT-PCR reactions, the qRT-PCR master mix was made by combining primers, SYBR select master mix, and nuclease-free water, followed by a brief vortexing of the mixture. qRT-PCR was performed in a Step-One Real-Time PCR system (Applied Biosystems). Two linked profiles, (1) one cycle of 50°C for 2 min and 95°C for 2 min, and (2) 40 cycles consisting of denaturation at 95°C for 15 s and annealing/extension at 60°C for 60 s, were used. Four to 6 biological replicates were performed for each data point. All the results corresponded to relative quantification using the *M. occidentalis actin* gene as an internal control using the 2^−ΔΔCt^ method [29]. Specifically, the Ct for each target (e.g. *clathrin heavy chain* gene) was subtracted by the Ct of *actin* gene from each sample (e.g. control dsRNA or *clathrin heavy chain* dsRNA) to produce ΔCt. The ΔCt from the control was then averaged to produce a mean ΔCt of the control. Then the mean ΔCt of the control was subtracted from individual ΔCt values from control or *clathrin heavy chain* dsRNA treated mites, to yield the ΔΔCt. Then ΔΔCt was used to produce the 2^−ΔΔCt^ estimates. The specificity of qRT-PCR was confirmed by melting-curve analyses after each reaction. In pilot experiments, the stability of 4 reference genes, *actin, TFIID*, *GAPDH*, and *RPS18* was evaluated in samples that received different dsRNAs. The a*ctin* gene was identified as the most stable reference gene with the use of BestKeeper [30] [data not shown].

### Statistical analysis

The means and standard errors of means (SEM) were analyzed by analysis of variance (ANOVA) (JMP 8; SAS Institute, Cary, NC), and means were separated using Tukey’s HSD test (*P*<0.05). For one-to-one comparisons, means and SEM were analyzed by ANOVA and means were separated by Student’s *t* test.

## Results and Discussion

### Identification of a putative *clathrin heavy chain* gene in the *Metaseiulus occidentalis* genome

**Figure 1:**
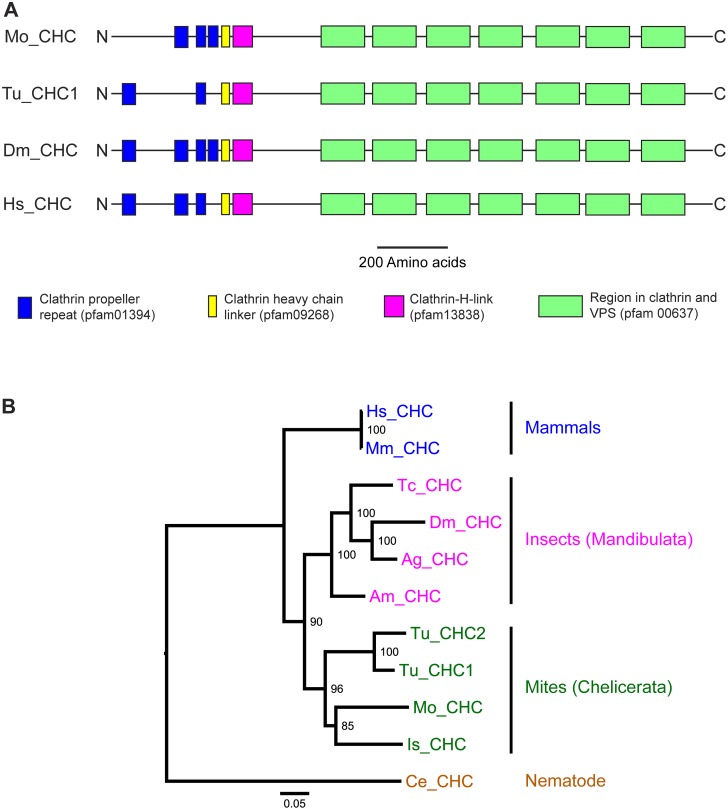
Domain structure and phylogenetic tree of clathrin heavy chain proteins of *M. occidentalis* and selected species. (**A**) Schematic structures of predicted clathrin heavy chain (CHC) proteins from the mites *M. occidentalis* (Mo) and *T. urticae* (Tu), the insect *D. melanogaster* (Dm), and the mammal *Homo sapiens* (Hs). Names and pfam ID of conserved domains are shown in brackets. (**B**) Phylogenetic analysis of predicted clathrin heavy chain proteins from selected species (Mo = *M. occidentalis*, Tu = *T. urticae*, Is = *I. scapularis*, Am = *A. mellifera*, Ag = *An. gambiae*, Tc = *T. castaneum*, Dm = *D. melanogaster*, Hs = *H. sapiens*, Ms = *M. musculus*, Ce = *C. elegans*). The tree was generated using a maximum likelihood approach [28] with bootstrap support values shown at the nodes. The tree was rooted using the clathrin heavy chain of the nematode *C. elegans* (Ce_CHC). The scale bar represents the numbers of substitutions per site.

The putative *M. occidentalis clathrin heavy chain* gene encodes a protein with 1686 amino acids, which is similar to the lengths of clathrin heavy chains from other species (Table 1). Moreover, the deduced *M. occidentalis* clathrin heavy chain protein sequence shares 70–85% identities with homologs from *Caenorhabditis elegans, Homo sapiens, D. melanogaster* and *T. urticae*. Figure 1A shows a comparison of the conserved domains in the *clathrin heavy chain* homologs of *M. occidentalis*, *T. urticae*, *D. melanogaster*, and *Homo sapiens*. The deduced *M. occidentalis* clathrin heavy chain protein has all the conserved domains found in the homologs of the other three species listed. The numbers and the relative positions of domains such as clathrin heavy chain linker, clathrin-H-link, and regions in clathrin and VPS are identical among all homologs listed. The number and relative positions of the clathrin propeller repeats show a slight variation among the listed homologs. Taken together, these data suggest that *clathrin heavy chain* genes are highly conserved at both the amino-acid sequence as well as the domain-composition levels across diverse species. Figure 1B shows the result of a phylogenetic analysis on *clathrin heavy chain* homologs based on an alignment of their amino-acid sequences (Fig. S1). The *M. occidentalis clathrin heavy chain* clusters with its homologs from other acarines (Arthropoda: Chelicerata) such as the tick *Ixodes scapularis* and the two-spotted spider mite *T. urticae*. As expected, *clathrin heavy chains* from insects (Arthropoda: Mandibulata) and mammals cluster with their respective groups.

**Table 1:** A list of clathrin heavy chain proteins in selected species.

Species	GenBankaccession number	Protein length(in amino acids)	Percentage identityto *M. occidentalis* clathrinheavy chain protein
*Metaseiulus occidentalis*	XP_003740941.1	1686	100
*Tetranychus urticae*	tetur01g04320*	1687	85
*Tetranychus urticae*	tetur03g00330*	1698	84
*Ixodes scapularis*	XP_002406240.1	1616	83
*Apis mellifera*	XP_623111	1678	82
*Anopheles gambiae*	XP_311856.3	1676	82
*Tribolium castaneum*	XP_967829.1	1684	82
*Drosophila melanogaster*	NP_477042.1	1678	80
*Homo sapiens*	NP_004850.1	1675	80
*Mus musculus*	NP_001003908.1	1675	80
*Caenorhabditis elegans*	NP_499260.1	1681	70

*Identifier for BOGAS database (http://bioinformatics.psb.ugent.be/orcae/overview/Tetur).

Percentage identity was determined by BLASTP searches.

### *Clathrin heavy chain* dsRNA delivery reduced longevity, oviposition, and embryogenesis

**Figure 2:**
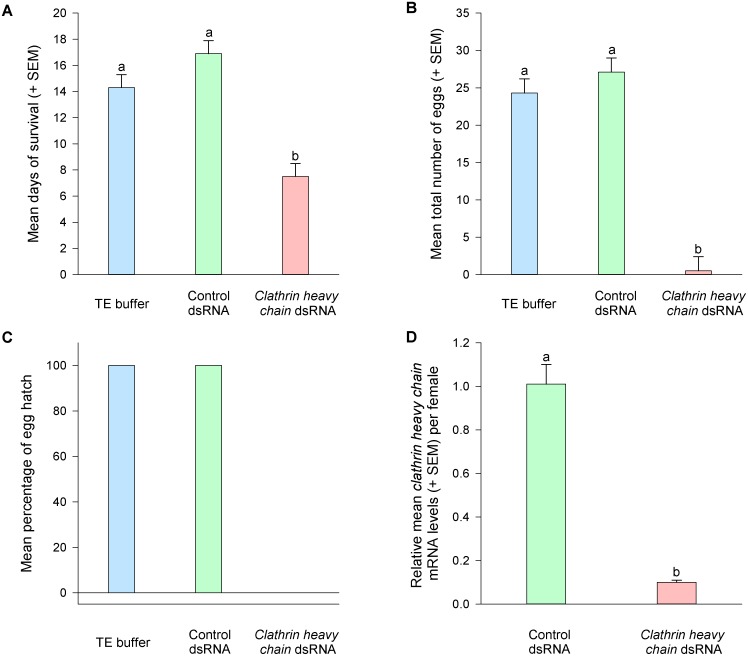
The effects of oral delivery of *clathrin heavy chain* dsRNA on survival and egg production of *M. occidentalis* females. Egg hatch rates and gene knockdown efficiency were also evaluated. (**A**) and (**B**) The effects of *clathrin heavy chain* dsRNA delivery on survival and oviposition of females, respectively. N = 10 for each treatment group. One-way ANOVA results for days of survival and number of eggs are *F_2,29_* = 20.55; *P*<0.0001, *F_2,29_* = 57.71; *P*<0.0001, respectively. Bars labeled with different letters are significantly different (Tukey’s HSD test, *P*<0.0004). (**C**) The effect of *clathrin heavy chain* dsRNA delivery on egg hatch rates. N = 10, 10, and 5 for TE buffer group, control dsRNA group, and *clathrin heavy chain* dsRNA group, respectively. (**D**) The effect of *clathrin heavy chain* dsRNA delivery on the mRNA levels of the *clathrin heavy chain* gene. N = 4 for each group. *Clathrin heavy chain* mRNA levels in control females were scaled to 1. Student’s *t* test result for the comparison of relative *clathrin heavy chain* mRNA levels between control dsRNA- and *clathrin heavy chain* dsRNA-treated mites is *P*<0.0001.

As shown in Fig. 2, oral delivery of *clathrin heavy chain* dsRNA into *M. occidentalis* females resulted in approximately 90% reduction in *clathrin heavy chain* mRNA levels when compared to controls (*clathrin heavy chain* vs. control, *P*<0.0001, *t* test). Females treated with *clathrin heavy chain* dsRNA exhibited survival rates about half of those that received control dsRNA or TE buffer (*clathrin heavy chain* dsRNA vs. control dsRNA or TE, *P*<0.0001, ANOVA, Tukey-Kramer HSD). Egg production was greatly reduced in females treated with *clathrin heavy chain* dsRNA. Half of the females did not produce any eggs and the other half produced only one egg per female. In contrast, all control females produced 24–27 eggs/female. Of the few eggs produced by females treated with *clathrin heavy chain* dsRNA none hatched, in contrast to 100% hatch rates for eggs produced by control females. The differences in the longevity and oviposition of *M. occidentalis* females that received control dsRNA or TE buffer were not significant. The egg hatch rates in both control groups were not significantly different.

These results support our hypothesis that clathrin heavy chain is essential for the viability and oviposition rate of *M. occidentalis* adult females and embryonic development. The results from the current study, and those from previous studies on *D. melanogaster* and *C. elegans* [11], [12], suggest that this highly conserved protein is likely essential for viability and embryonic development in multicellular organisms, including some arthropods (Table 1).

### *Clathrin heavy chain* gene knockdown severely impaired a subsequent RNAi response

**Figure 3:**
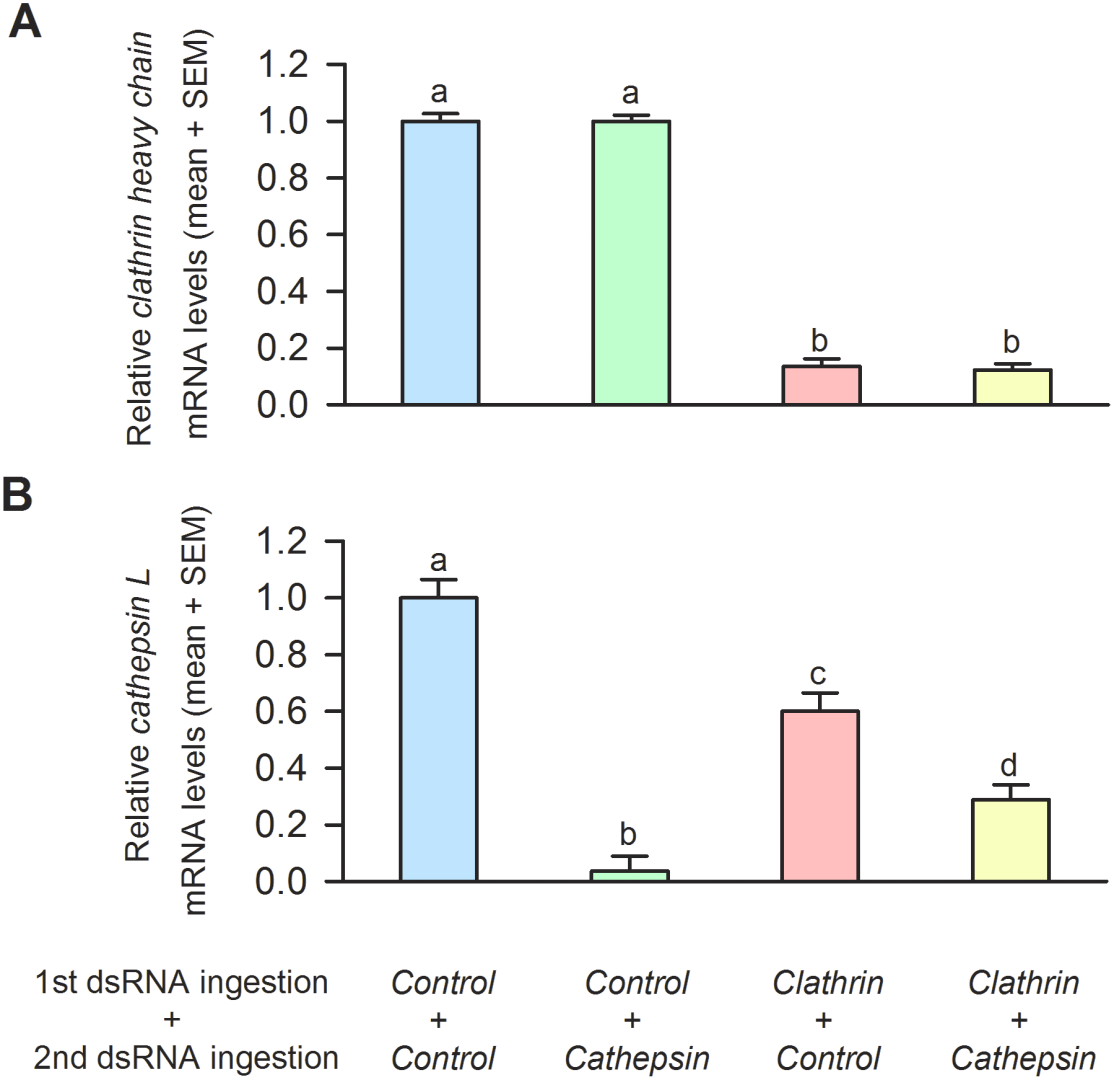
The effects of *clathrin heavy chain* gene knockdown on a subsequent RNAi response as measured by *cathepsin L* knockdown in *M. occidentalis* females. (**A**) The effects of different treatment combinations on the relative mRNA levels of *clathrin heavy chain* (mean + SEM). Ingestion of *clathrin heavy chain* dsRNA resulted in a reduction of *clathrin heavy chain* mRNA levels in *Clathrin + Control* and *Clathrin + Cathepsin* mites. (**B**) The effect of different treatment combinations on the relative mRNA levels of *cathepsin L* (mean + SEM). *Clathrin heavy chain* dsRNA pretreatment reduced subsequent RNAi responses as evaluated by gene knockdown of *cathepsin L*. For *clathrin heavy chain* and *cathepsin L* mRNAs comparisons: One-way ANOVA, *F_3,19_* = 470.10, *P*<0.0001 and *F_3,19_* = 49.16, P<0.0001, respectively, Tukey-Kramer HSD lettering for all comparisons. The mRNA levels of *clathrin heavy chain* and *cathepsin L* in *Control + Control* group are scaled to 1. Different letters denote significant differences.

To investigate whether *clathrin heavy chain* gene knockdown affected subsequent systemic RNAi responses, *M. occidentalis* females were fed with control or *clathrin heavy chain* dsRNA first (pretreatments or first ingestion). These females were then provided with spider mite prey, which is required to initiate gene knockdown after ingestion of dsRNA [21]. Next, these females were fed control or *cathepsin L* dsRNA (second ingestion; for a detailed description of the experimental design, see Table S1). *Cathepsin L* gene knockdown was chosen as a marker for measuring systemic RNAi potency because the *cathepsin L* gene could be efficiently knocked down (for details, see below and Fig. 3B, *Control + Cathepsin* vs. *Control + Control*). In addition, *cathepsin L* gene knockdown had no discernible effect on the viability of *M. occidentalis* females [Wu and Hoy, unpublished]. Therefore, the use of *cathepsin L* knockdown as a marker for RNAi potency likely ensured more mites survived the subsequent assays. Fig. 3A illustrates the relative *clathrin heavy chain* mRNA levels in the treatment groups. The *clathrin heavy chain* mRNA levels in *Control + Cathepsin* mites were not significantly different from those in *Control + Control* mites, indicating that *cathepsin L* dsRNA treatment had no effect on *clathrin heavy chain* mRNA level. In contrast, *clathrin heavy chain* mRNA levels in *Clathrin + Control* mites were 13.6% of those of *Control + Control* mites, indicating that *clathrin heavy chain* dsRNA induced a robust gene knockdown (i.e. knockdown efficiency of 86.4%). Similarly, *clathrin heavy chain* mRNA levels in *Clathrin + Cathepsin* mites were 11.3% of those of *Control + Cathepsin* mites, indicating that the robust gene knockdown induced by *clathrin heavy chain* dsRNA was not affected by the subsequent feeding of *cathepsin L* dsRNA.

Fig. 3B shows the relative mRNA levels of *cathepsin L* in the different treatment groups. In mites pretreated with control dsRNA, subsequent *cathepsin L* dsRNA treatment induced a 96.4% gene knockdown (*Control + Control* vs *Control + Cathepsin*). In mites pretreated with *clathrin heavy chain* dsRNA, subsequent *cathepsin L* dsRNA treatment only achieved a modest 52.5% gene knockdown (*Clathrin + Control* vs. *Clathrin + Cathepsin*). Taken together, these results indicate that *clathrin heavy chain* dsRNA pretreatment greatly reduced systemic RNAi potency as assayed by *cathepsin L* dsRNA gene knockdown. These findings support our hypothesis that clathrin heavy chain plays an important role in the systemic RNAi response in *M. occidentalis*. We included the *Clathrin + Control* group as a control on which the calculation of the reporter gene knockdown efficiency in *clathrin heavy chain* dsRNA-treated mites was based to control for any effect the *clathrin heavy chain* dsRNA pretreatment might have had on the expression of the c*athepsin L* gene. Indeed, *clathrin heavy chain* dsRNA treatment, by itself (*Clathrin + Control* mites), reduced the *cathepsin L mRNA* levels by 40%, when compared to *Control + Control* mites, indicating that *clathrin heavy chain* gene knockdown likely had a broad- spectrum effect that affected the transcriptional regulation of other genes.

In our assays, *clathrin heavy chain* dsRNA pretreatment did not completely abolish the subsequent RNAi response mediated by *cathepsin L* dsRNA. This is likely due to an incomplete *clathrin heavy chain* knockdown (∼87%, Fig. 3A). In addition, clathrin heavy chains are known to be relatively stable [31]; therefore it was possible that a significant amount of clathrin heavy chain remained when *cathepsin L* dsRNA uptake occurred, thus resulting in a modest, though significant, *cathepsin L* gene knockdown (*Clathrin + Cathepsin* mites vs. *Clathrin + Control* mites, Fig. 3B).

We previously showed that ingestion of dsRNA elicited robust and systemic RNAi responses in *M. occidentalis* [21]. One discovery from that study was that *M. occidentalis* females did not initiate RNAi responses after ingesting dsRNA in 20% sucrose for 3 days unless the dsRNA/sucrose solution was replaced by *T. urticae* prey. It is possible that components in the spider mite prey are required to initiate an RNAi response in *M. occidentalis*. Another possibility, not necessarily mutually exclusive with the first one, is that factors in the dsRNA +20% sucrose inhibit RNAi responses, possibly at the dsRNA uptake step. Hypertonic media (e.g., 0.45 M sucrose in normal media) have been shown to inhibit receptor-mediated endocytosis by blocking the formation of clathrin-coated pits in mammalian cell cultures [32]. This inhibition could be reversed by the removal of high-concentration sucrose [32]. The 20% sucrose concentration in our dsRNA solution is equivalent to 0.58 M. Before they were fed dsRNA in sucrose, *M. occidentalis* females were starved for 24 h, which likely reduced the liquid content in their guts, as indicated by the flattening of their bodies [Wu and Hoy, unpublished]. Therefore, it was possible that sucrose concentrations remained high in the guts of *M. occidentalis* females after they ingested dsRNA in 20% sucrose solution and high concentrations of sucrose could have blocked the clathrin-mediated endocytosis of dsRNA by the gut cells. After replacing the dsRNA in sucrose with *T. urticae* prey, the sucrose concentrations in the guts of *M. occidentalis* likely decreased and the clathrin-mediated endocytosis of dsRNA likely became derepressed. Thus, our previous observation is consistent with the notion that the clathrin heavy chain is involved in systemic RNAi responses in *M. occidentalis*. However, it is not clear whether clathrin heavy chain is involved in the initial uptake of dsRNA by cells in the gut lumen and/or in the subsequent uptake in the peripheral tissues. Finally, because the knockdown of *clathrin heavy chain* apparently induced a general deleterious effect on *M. occidentalis*, we cannot exclude the possibility that the subsequently reduced RNAi response might be a result of a nonspecific/toxic effect that was caused by the knockdown of this important protein.

Our finding that clathrin heavy chain may be involved in RNAi in *M. occidentalis*, together with similar results from *D. melanogaster* and *S. gregaria* [13], [14], [15], lends support to the hypothesis that clathrin heavy chain may be involved in RNAi responses in all arthropods. Two scavenger receptors were identified that were responsible for the majority of dsRNA uptake in *D. melanogaster* S2 cells [14]. It is likely that scavenger receptors also play a role in the RNAi machinery in *M. occidentalis*. Future studies are needed to identify the receptors involved in the RNAi response in *M. occidentalis*.

In summary, we show that the conserved clathrin heavy chain is critical for survival and egg production in adult females of *M. occidentalis*, as well as viability of the few embryos produced. Clathrin heavy chain also may be involved in the systemic RNAi response in this mite. The current study represents, to our knowledge, only the second study in which a detailed loss-of-function analysis has been performed on clathrin heavy chain in a multicellular organism *in vivo*. Our findings support the notion that this protein is important for the survival, embryonic development, and, possibly, RNAi responses in multicellular organisms.

## Supporting Information

Figure S1 A multiple sequence alignment file (in FASTA format) of the deduced amino-acid sequences of *clathrin heavy chain* (CHC) genes from the mites *M. occidentalis* (Mo), *I. scapularis* (Is) and *T. urticae* (Tu), the insects *T*. *castaneum* (Tc), *A*. *mellifera* (Am), *An. gambiae* (Ag) and *D. melanogaster* (Dm), and the mammals *H. sapiens* (Hs) and *M. musculus* (Mm), and the nematode *C. elegans* (Ce).
(DOCX)

Table S1 The experimental design for the study on the effects of *clathrin heavy chain* gene knockdown on subsequent RNAi responses in *Metaseiulus occidentalis*.
(DOCX)
