# Supplementary material for: Clathrin Heavy Chain Is Important for Viability, Oviposition, Embryogenesis and, Possibly, Systemic RNAi Response in the Predatory Mite Metaseiulus occidentalis
Source: PLoS One. 2014 Oct 20;9(10):e110874. doi: 10.1371/journal.pone.0110874 (PMC4203830; doi:10.1371/journal.pone.0110874)
Supplement: Figure S1 — A multiple sequence alignment file (in FASTA format) of the deduced amino-acid sequences of clathrin heavy chain (CHC) genes from the mites M. occidentalis (Mo), I. scapularis (Is) and T. urticae (Tu), the insects T. castaneum (Tc), A. mellifera (Am), An. gambiae (Ag) and D. melanogaster (Dm), and the mammals H. sapiens (Hs) and M. musculus (Mm), and the nematode C. elegans (Ce). (DOCX) [file pone.0110874.s001.docx]

>Mo_CHC

MAQTLPIKFQEHLQLTSVGINAATIGFNTLTMESDKYICVREKIGDSNHVVIIDMATPQQPIRRPISADSAIMNPASKVIALK------ANRTLQIFNIEMKSKMKAHAMSEDVVFWKWINLNTLALVTESAVYHWSMEGDSQPVKMFDRHSSLASCQIINYRTDHKLQWLLLVGISAQQNRVVGAMQLYSMERKVSQPIEGHAAAFAQLEVEGNSQPSTIFCFAVRTQAGGKLHLVEVGTPAPGNQPFSKKGIDVFFPPEAQSDFPVAMQVSPRHDVVYLITKYGYVHLYDLESGTCIYMNRISSDTIFVTAPHEASSGIIGVNRKGQVLSVSVDEENIVNYIMTTLNNPDLALRIASRNNLPGADDLFVHKFNNLFQSGQYTEAAKVAANAPKGILRTPQTIQRFQQVPQQPGQTT-PLLQYFGILLDKGKLNKFESLELCRPVLQQGRKQLLEKWLKDEKLECSEELGDLVKQVDPTLALAVYLRANVPPKVIQCFAETGQFQKIVLYAKKVGYTPDYIQILRQVMR-VAPDQGPVFAQMLVADG--EQPLADVAQIVDVFMEANLVQQCTAFLLDALKHNRPTEGALQTRLLEMNLIAAPQVADAILGNQMFSHYDKAHIAQLCEKANLLQRALEHYTDLYDIKRAIVNTHLLNADWLVNYFGSLSVEDSLECLKAMLSHNIRQNLQICVQVATKYHEQLTTVALIELFESFKSYEGLFYFLGSIVNFSQDPEVHFKYIQAACKTGQIKEVERICRESNCYNAERVKNFLKEAKLTDQLPLIIVCDRFDFVHDLVLYLYRNNLQKYIEIYVQKVNPSRLPVVVGGLLDVDCSEDVIKSLIIAVRGGQFSTDELVDEVEKRNRLKLLLPWLETRVHEGCTEPATHNALAKIYIDSNN

>Is_CHC

MTQMLPIRFQEHLQLTNIGINAANVGFNTLTMESDKFICVREKVGDAAQVVIVDMANPTSPIRRPISADSAIMNPASRVIALK------ASRTLQIFNIEMKSKVKAHTMTEDVVFWKWINVNTIALVTEGAVYHWSMEGDSQPQKMFDRHSSLSGCQIINYRTDAKIQWLLLIGISAQQNRVVGAMQLYSMERKVSQPIEGHAAAFAQFKQEGNTEASTLFCFAVRTPHGGKLHIIEVGQPAAGNQAYPKKAVDVFFPPEAQNDFPVAMQMSPRHDVVYLITKYGYVHLYDLESGTCIYMNRISADTIFVTAPHEASSGIIGVNRKGQVLSVSVEEENIIPYITNVLQNPDLALRMAVRNNLAGAEDLFVVKFNTLFGGGQYSEAAKVAANAPKGILRTPQTIQRFQQVPNQPGQTS-PLLQYFGILLDQGQLNKYESLELCRPVLQQGRKQLLEKWLKDDKLECSEELGDLVKQVDPTLALSVYLRANVPNKVIQCFAETGQFQKIVLYAKKVGYVPDYVLLLRQVMR-VNPDQGASFAQMLVQD---EEPLADINQIVDVFMESNLVQQCTAFLLDALKNNRPSESNLQTRLLEMNLMTAPQVADAILGNQMFTHYDRAHVAQLCEKAGLLQRALEHYTDLYDIKRAIVHTHLLNAEWLVNYFGSLSVEDSLECLRAMLTHNLRQNLQISVQVATKYHEQLTTASLIDLFESFKSYEGLFYFLGSIVNFSQDPEVHFKYIQAACKTGQIKEVERICRESNCYNAERVKNFLKEAKLTDQLPLIIVCDRFDFVHDLVLYLYRNSLQKYIEIYVQKVNPSRLPVVVGGLLDVDCAEEVIKGLILVVR-GQFSTDELVAEVEKRNRLKLLLPWLEGRLHEGCQEPATHNALAKIYIDSNN

>Tu_CHC1

MSQTLPIKFQEHLQLTAIGINAANITFNTLTMESDKFICVREKVGDSAQVVIIDMSNPTTPIRRPISADSAIMNPASKVIALK------AMRTLQIFNIEMKSKMKAHTMTEDVVFWKWINLNTIALVTEGAVYHWSMEGDSQPVKMFDRHASLQGCQIINYRTDHRVQWLLLIGISASQNRVVGAMQLYSTERKVSQPIEGHAAAFSKFKMEGNSEPSTLFCFAARTAQGGKLHIIEVGQPPTGNQPYPKKAVDVFFPAEAQNDFPVAMQISTKYDIIYLITKFGYVHLYDIETGICIYMNRITGDTIFVTAPYEPTSGIIGVNRKGQVLSVCVDEENIIPYITTTLANPDLALRIAARNNLPGAEDLFVRKFNTLFANSQYTEAAKVAASAPKGILRTPETIRRFQQVPTQPGQTS-PLLQYFGILLDHFQLNKFESLELCRPVLQQGRKQLLEKWLKDDKLECSEELGDLVKAVDPTLALSVYLRANVPNKVIQCFAETGQFQKIVLYAKKVGYTPDYTNLLRQVMR-TNPDQGAAFAQMLVQD---EEPLSDINQIVDVFMESNLVQPCTVFLLEALKHNRPTEGPLQTRLLEMNLVTAPQVADAILGNGVFTHYDRAHVAQLCEKAGLLQRALEHYTDLYDIKRAIVHTHLLNPEWLVNYFGTLSVEDSIECLKAMLTHNIRQNLQIAVQVATKYHEQLTTTSLIELFESFKSYEGLFYFLGSIVNFSQDPEVHFKYIQAACKTGQIKEVERICRESNCYNAERVKNFLKEAKLTDQLPLIIVCDRFDFVHDLVLYLYRNNLQKYIEIYVQKVNPSRLPVVIGGLLDVDCSEDVIKQLIMVVK-GQFSTDELVEEVEKRNRLKLLLPWLEMRVHEGCTEAATHNALAKIYIDSNN

>Tu_CHC2

MSQSLPIKFQEHLQLTAIGINAANITFNTLTMESDKFICVREKVGDSAQVVIIDMSNPTTPIRRPISADSAIMNPASKVIALK------AMRTLQIFNIEMKSKMKAHTMADDVVFWKWINVNTIALVTETAVYHWSMEGDSQPVKMFDRHASLTGCQIINYRTDHRVQWLLLIGISAAQGRVVGAMQLYSTERKVSQPIEGHAAAFSTFKMDGNSEVSTLFCFAARTSTGGKLHIIEVGQPPAGNQPYPKKAVDVFFPTEAQNDFPVAMQVSTKYDIIYLITKYGYIHLYDIETGTCIYMNRISGDTIFVTAPYEPTSGIIGVNRKGQVLSVCVDEDAIIPYITTTLANPDLALRIAARNNLPGAEELFVRKFNTLFSSGQYTEAAKAAANAPKGILRTPDTIRRFQQVPTQPGQTS-PLLQYFGILLDHFQLNKYESLELCRPVLQQGRKQLLEKWLKDDKLECSEELGDLVKAVDPTLALSVYLRANVPNKVIQCFAETGQFQKIVLYAKKVGYTPDYSNLLRQVMR-TNPDQGAAFAQMLVQD---EEPLANINQIVDVFMESNLVQPCTVFLLEALKNNRPEEGPLQTRLLEMNLVTAPQVADAILGNGVFTHYDRPHIASLCEKAGLLQRALEHYSDLYDIKRAIVHTHLLNTEWLVNYFGTLSVEDSLECIKAMLQHNIRQNLQIAVQVATKYHEQLGTTALIELFESFKSYEGLFYFLGSIVNFSQDPDVHFKYIQAACKTGQIKEVERICRQSNCYNAERVKNFLKEAKLTDQLPLIIVCDRFDFVHDLVLYLYRNNLQKYIEIYVQKVNPSRLPVVIGGLLDVDCSEDVIKSLMLVVK-GQFSTDELVEEVEKRNRLKLLLPWLENRVHEGCTEPATHNALAKIYIDSNN

>Tc_CHC

TQQLLPIKFQEHLQLTNVGINVANISFATLTMESDKFICVREKVGDTSQVVIIDMGDTANPIRRPITAESAIMNPASKVIALKGKAGVEAQKTLQIFNIEMKSKMKAHTMSEDVIFWKWISLNTLALVTETSVYHWSMEGDSTPVKMFDRHSSLNGCQIINYRTDPKQNWLLLVGISAQQSRVVGAMQLYSVERKCSQPIEGHAASFATFKMEGNPEPSTLFCFAVRTVQGGKLHIIEVGQSPAGNQPFPKKTVDVFFPPEAQNDFPVAMQVSAKYDVIYLITKYGYIHMYDIESAICIYMNRISSETIFVTAPHESTGGIIGVNRRGQVLSVSVDEDSIIRYVNQVLHNPDLALRIATRNNLAGAEELFVNKFQMLFTNGQYAEAAKVAANAPKGILRTPATIQMFQQVPTQPGQNS-PLLQYFGILLDQGQLNRYESLELCKPVLLQGRKQLLEKWLKEDKLECSEELGDLVKQADSTLALSVYLRANVPAKVIQSFAETGQFQKIVLYAKKVNYTPDYIYLLRSVMR-TNPDQGAAFASMLVAD---EEPLADINQIVDIFMEQNMVQQCTAFLLDALKHNRPTEGHLQTRLLEMNLMSAPQVADAILGNNMFTHYDRAHIAQLCEKAGLLQRALEHYTDLYDIKRAVVHTHLLPMDWLVNFFGTLSVEDSLECLKAMLTANIRQNLQICVQIATKYHEQLTTKALIDLFESFKSYEGLFYFLGSIVNFSQDPDVHFKYIQAACKTGQIKEVERICRESNCYNPESVKNFLKEAKLTDQLPLIIVCDRFDFVHDLVLYLYRNSLQKYIEIYVQKVNPSRLPVVVGGLLDVDCAEDIIKNLILVVR-GQFSTDELVEEVEKRNRLKLLLPWLESRVHEGCVEPATHNALAKIYIDSNN

>Am_CHC

MTQLLPIRFQEHLQLTAVGINANNVSFNTLTMESDKFICVREKVGDTAQVVIIDMNDSANPIRRPISADSAIMNPASKVIALK------AMKTLQIFNIEMKSKMKAHTMTEDVVFWKWISLNTLALVTETAVYHWSMEGESTPNKMFDRHSSLNGCQIINYRTDPKQTWLLLIGISAQHNRVVGAMQLYSVERKCSQPIEGHAASFAQFKMEGNAEPSNLFCFAVRTVQGAKLHIIEVGQPPAGNHPFPKKAVDVFFPPEAGNDFPVAMQVSSKYDVIYLITKYGYIHMYDIESATCIFMNRISGETIFVTAPHEASGGIIGVNRKGQVLSVSVDEENIIPYINGVLQNSELALRMAVRNNLSGAEDLFVRKFNLLFQNGQYAEAAKVAANAPKGILRTPATIQRFQQVPTTQGQTS-PLLQYFGILLDQGQLNKYESLELCRPVLVQGRKQLLEKWLKEDKLECSEELGDLVKQADPTLALSVYLRANVPNKVIQCFAETGQFQKIVLYAKKVSYTPDYIFLLRNVMR-INPDQGVAFAQMLVQD---DEPLADINQIVDIFMEQNMVQQCTAFLLDALKNNRPSEGALQTRLLEMNLMSAPQVADAILGNQMFTHYDRAHIAQLCEKAGLLQRALEHYTDLYDIKRAVVHTHLLSPDWLVGFFGTLSVEDSLECLKAMLTANIRQNLQICIQIATKYHEQLTTKALIDLFESFKSYEGLFYFLGSIVNFSQDQEVHFKYIQAACKTGQIKEVERICRESNCYNPERVKNFLKEAKLSDQLPLIIVCDRFDFVHDLVLYLYRNNLQKYIEIYVQKVNPSRLPVVVGGLLDVDCSEDIIKNLILVVR-GQFSTDELVEEVEKRNRLKLLLPWLESRVHEGCVEPATHNALAKIYIDSNN

>Ag_CHC

MSQQLPIRFQEHLQLTNININASSISFTNLTMESDKFICVREKVGETAQVVIIDMNDAQNPIRRPISADSAIMNPASKVIALK------AQKTLQIFNIEMKSKMKAHTMTEEVVFWKWITLNTLSLVTETSVYHWSMEGDSTPIKMFERHSSLNGCQIINYRTDPKQAWLLLVGISAQQNRVIGAMQLYSVERKVSQAIEGHAASFATFKMEENKELSTLFCFAVRSQTAAKLHIIEVGTPPAGNVAFTKKAVDVFFPPEAQSDFPVAMQVSPRYDVIYLITKYGYIHMYDIETATCIYMNRISGDTIFVTAPHESSGGIIGVNRKGQVLSVTVDEEQIIPYINTVLQNPDLALRMAVRNNLSGAEDLFVRKFNQLFQNGQFAEAAKVAAIAPKGILRTPQTIQKFQQVPAQPGTNSPPLLQYFGILLDQGKLNKYESLELCRPVLAQGRKQLCEKWLKEEKLECSEELGDLVKPSDPTLALSIYLRSNVPNKVIQCFAETGQFQKIVLYAKKVNYSPDYVFLLRSVMR-TNPEQGSGFASMLVAD---EEPLADINQIVDIFMEQNMVQQCTAFLLDALKNNRPAEGALQTRLLEMNLMSAPQVADAILGNAMFTHYDRAHIAQLCEKAGLLQRALEHYTDLYDIKRAVVHTQLLNGDWLVGFFGTLSVEDSLECLKAMLTANIRQNLQICVQIATKYHEQLTTKALIDLFESFKSYEGLFYFLGSIVNFSQDPEVHFKYIQAACKTNQIKEVERICRESNCYNAERVKNFLKEAKLTDQLPLIIVCDRFDFVHDLVLYLYRNSLQKYIEIYVQKVNPSRLPVVVGGLLDVDCSEDIIKNLILVVK-GQFSTDELVEEVEKRNRLKLLLPWLESRVHEGCVEPATHNALAKIYIDSNN

>Dm_CHC

MTQPLPIRFQEHLQLTNVGINANSFSFSTLTMESDKFICVREKVNDTAQVVIIDMNDATNPTRRPISADSAIMNPASKVIALK------AQKTLQIFNIEMKSKMKAHTMNEDVVFWKWISLNTLALVTETSVFHWSMEGDSMPQKMFDRHSSLNGCQIINYRCNASQQWLLLVGISALPSRVAGAMQLYSVERKVSQAIEGHAASFATFKIDANKEPTTLFCFAVRTATGGKLHIIEVGAPPNGNQPFAKKAVDVFFPPEAQNDFPVAMQVSAKYDTIYLITKYGYIHLYDMETATCIYMNRISADTIFVTAPHEASGGIIGVNRKGQVLSVTVDEEQIIPYINTVLQNPDLALRMAVRNNLAGAEDLFVRKFNKLFTAGQYAEAAKVAALAPKAILRTPQTIQRFQQVQTPAGSTTPPLLQYFGILLDQGKLNKFESLELCRPVLLQGKKQLCEKWLKEEKLECSEELGDLVKASDLTLALSIYLRANVPNKVIQCFAETGQFQKIVLYAKKVNYTPDYVFLLRSVMR-SNPEQGAGFASMLVAE---EEPLADINQIVDIFMEHSMVQQCTAFLLDALKHNRPAEGALQTRLLEMNLMSAPQVADAILGNAMFTHYDRAHIAQLCEKAGLLQRALEHYTDLYDIKRAVVHTHMLNAEWLVSFFGTLSVEDSLECLKAMLTANLRQNLQICVQIATKYHEQLTNKALIDLFEGFKSYDGLFYFLSSIVNFSQDPEVHFKYIQAACKTNQIKEVERICRESNCYNPERVKNFLKEAKLTDQLPLIIVCDRFDFVHDLVLYLYRNNLQKYIEIYVQKVNPSRLPVVVGGLLDVDCSEDIIKNLILVVK-GQFSTDELVEEVEKRNRLKLLLPWLESRVHEGCVEPATHNALAKIYIDSNN

>Hs_CHC

MAQILPIRFQEHLQLQNLGINPANIGFSTLTMESDKFICIREKVGEQAQVVIIDMNDPSNPIRRPISADSAIMNPASKVIALK------AGKTLQIFNIEMKSKMKAHTMTDDVTFWKWISLNTVALVTDNAVYHWSMEGESQPVKMFDRHSSLAGCQIINYRTDAKQKWLLLTGISAQQNRVVGAMQLYSVDRKVSQPIEGHAASFAQFKMEGNAEESTLFCFAVRGQAGGKLHIIEVGTPPTGNQPFPKKAVDVFFPPEAQNDFPVAMQISEKHDVVFLITKYGYIHLYDLETGTCIYMNRISGETIFVTAPHEATAGIIGVNRKGQVLSVCVEEENIIPYITNVLQNPDLALRMAVRNNLAGAEELFARKFNALFAQGNYSEAAKVAANAPKGILRTPDTIRRFQSVPAQPGQTS-PLLQYFGILLDQGQLNKYESLELCRPVLQQGRKQLLEKWLKEDKLECSEELGDLVKSVDPTLALSVYLRANVPNKVIQCFAETGQVQKIVLYAKKVGYTPDWIFLLRNVMR-ISPDQGQQFAQMLVQD---EEPLADITQIVDVFMEYNLIQQCTAFLLDALKNNRPSEGPLQTRLLEMNLMHAPQVADAILGNQMFTHYDRAHIAQLCEKAGLLQRALEHFTDLYDIKRAVVHTHLLNPEWLVNYFGSLSVEDSLECLRAMLSANIRQNLQICVQVASKYHEQLSTQSLIELFESFKSFEGLFYFLGSIVNFSQDPDVHFKYIQAACKTGQIKEVERICRESNCYDPERVKNFLKEAKLTDQLPLIIVCDRFDFVHDLVLYLYRNNLQKYIEIYVQKVNPSRLPVVIGGLLDVDCSEDVIKNLILVVR-GQFSTDELVAEVEKRNRLKLLLPWLEARIHEGCEEPATHNALAKIYIDSNN

>Mm_CHC

MAQILPIRFQEHLQLQNLGINPANIGFSTLTMESDKFICIREKVGEQAQVVIIDMNDPSNPIRRPISADSAIMNPASKVIALK------AGKTLQIFNIEMKSKMKAHTMTDDVTFWKWISLNTVALVTDNAVYHWSMEGESQPVKMFDRHSSLAGCQIINYRTDAKQKWLLLTGISAQQNRVVGAMQLYSVDRKVSQPIEGHAASFAQFKMEGNAEESTLFCFAVRGQAGGKLHIIEVGTPPTGNQPFPKKAVDVFFPPEAQNDFPVAMQISEKHDVVFLITKYGYIHLYDLETGTCIYMNRISGETIFVTAPHEATAGIIGVNRKGQVLSVCVEEENIIPYITNVLQNPDLALRMAVRNNLAGAEELFARKFNALFAQGNYSEAAKVAANAPKGILRTPDTIRRFQSVPAQPGQTS-PLLQYFGILLDQGQLNKYESLELCRPVLQQGRKQLLEKWLKEDKLECSEELGDLVKSVDPTLALSVYLRANVPNKVIQCFAETGQVQKIVLYAKKVGYTPDWIFLLRNVMR-ISPDQGQQFAQMLVQD---EEPLADITQIVDVFMEYNLIQQCTAFLLDALKNNRPSEGPLQTRLLEMNLMHAPQVADAILGNQMFTHYDRAHIAQLCEKAGLLQRALEHFTDLYDIKRAVVHTHLLNPEWLVNYFGSLSVEDSLECLRAMLSANIRQNLQICVQVASKYHEQLSTQSLIELFESFKSFEGLFYFLGSIVNFSQDPDVHFKYIQAACKTGQIKEVERICRESNCYDPERVKNFLKEAKLTDQLPLIIVCDRFDFVHDLVLYLYRNNLQKYIEIYVQKVNPSRLPVVIGGLLDVDCSEDVIKNLILVVR-GQFSTDELVAEVEKRNRLKLLLPWLEARIHEGCEEPATHNALAKIYIDSNN

>Ce_CHC

--MALPIKFHEHLQLPNAGIRVPNITFSNVTMESDKNIVVREMIGDQQQVVIIDLADTANPTRRPISADSVIMHPTAKILALK------SGKTLQIFNIELKAKVKAHQNVEDVVYWKWISEKTIALVSDTAVYHWSIEGDAAPVKMFDRHQSLAGTQIINYRADAENKWLVLIGISAKDSRVVGSMQLYSTERKVSQPIEGHAACFVRFKVDGNQNPSNLFCFSVKTDNGGKLHVIEVGTPAAGNTPFQKKNVDVPYTADTAGDFPVSMQVSAKQGIIYLVTKQGYVHLYDVESGTRIYSNRISTDTVFVTCEYTATGGIMGINRKGQVLSVSIDEANLVPFVTNQLQNPDLALKLAVRCDLPGAEELFVRKFNLLFSNGQFGESAKVAASAPQGILRTPATIQKFQQCPSTGPGPS-PLLQYFGILLDQGKLNKYETLELCRPVLAQGRKELITKWLNDQKLECCEELGDLIKPHDVNTALSVYLRGNVPHKVVQSFAETGQFDKIVMYAKRVGFQPDYLFQLRQILRNSNPDHGAKFAQLLVSESENGEPLADLSQIIDCFMEVQAVQPCTSFLLEVLKGDKPEEGHLQTRLLEMNLLAAPAVADAILANKMFSHYDRAAIGQLCEKAGLLQRALEHFTDLYDIKRTVVHTHLLKPDWLVGYFGSLSVEDSVECLKAMLTQNIRQNLQVVVQIASKYHEQLGADKLIEMFENHKSYEGLFYFLGSIVNFSQDPEVHFKYIQAATRTGQIKEVERICRESQCYDAERVKNFLKEAKLNDQLPLIIVCDRHNMVHDLVLYLYRNQLQKYIEVFVQKVNAARLPIVVGALLDVDCSEDAIKQLIINTR-GKFDIDELVEEVEKRNRLKLLNHWLESKIQEGATDAATHNAMAKIYIDSNN
