# Supplementary material for: Clathrin Heavy Chain Is Important for Viability, Oviposition, Embryogenesis and, Possibly, Systemic RNAi Response in the Predatory Mite Metaseiulus occidentalis
Source: PLoS One. 2014 Oct 20;9(10):e110874. doi: 10.1371/journal.pone.0110874 (PMC4203830; doi:10.1371/journal.pone.0110874)
Supplement: Table S1 — The experimental design for the study on the effects of clathrin heavy chain gene knockdown on subsequent RNAi responses in Metaseiulus occidentalis. (DOCX) [file pone.0110874.s002.docx]

| Treatment groups | N | dsRNA used for the first ingestion in 20% sucrose | dsRNA used for the second ingestion in 20% sucrose |
| --- | --- | --- | --- |
| *Control + Control* | 4 | Control dsRNA | Control dsRNA |
| *Control + Cathepsin* | 6 | Control dsRNA | Cathepsin L dsRNA |
| *Clathrin + Control* | 4 | *Clathrin heavy chain dsRNA* | Control dsRNA |
| *Clathrin + Cathepsin* | 6 | *Clathrin heavy chain dsRNA* | Cathepsin L dsRNA |

The first and second dsRNA ingestions were carried out with a 120-hr interval between them.
